# Naturally occurring cancer-associated mutations disrupt oligomerization and activity of protein arginine methyltransferase 1 (PRMT1)

**DOI:** 10.1016/j.jbc.2021.101336

**Published:** 2021-10-22

**Authors:** Owen M. Price, Abhishek Thakur, Ariana Ortolano, Arianna Towne, Caroline Velez, Orlando Acevedo, Joan M. Hevel

**Affiliations:** 1 Department of Chemistry and Biochemistry, Utah State University, Logan, Utah, USA; 2 Department of Chemistry, University of Miami, Coral Gables, Florida, USA

**Keywords:** arginine methylation, enzyme mutation, molecular dynamics, oligomerization, posttranslational modification (PTM), structure–function, signaling, dimerization, protein arginine methyltransferase, PRMT1, AdoMet, S-adenosyl-L-methionine, ADMA, asymmetric dimethylarginine, aMD, accelerated molecular dynamics, AUC, analytical ultracentrifugation, COSMIC, Catalogue of Somatic Mutations in Cancer, hPRMT1, H. *sapiens* PRMT1, MD, molecular dynamics, MMA, monomethylarginine, NAC, near attack conformation, PRMT, protein arginine methyltransferase, QM, quantum mechanics, rPRMT1, R. *norvegicus* PRMT1, SDMA, symmetric dimethylarginine, SV, sedimentation velocity

## Abstract

Protein arginine methylation is a posttranslational modification catalyzed by the protein arginine methyltransferase (PRMT) enzyme family. Dysregulated protein arginine methylation is linked to cancer and a variety of other human diseases. PRMT1 is the predominant PRMT isoform in mammalian cells and acts in pathways regulating transcription, DNA repair, apoptosis, and cell proliferation. PRMT1 dimer formation, which is required for methyltransferase activity, is mediated by interactions between a structure called the dimerization arm on one monomer and a surface of the Rossman Fold of the other monomer. Given the link between PRMT1 dysregulation and disease and the link between PRMT1 dimerization and activity, we searched the Catalogue of Somatic Mutations in Cancer (COSMIC) database to identify potential inactivating mutations occurring in the PRMT1 dimerization arm. We identified three mutations that correspond to W215L, Y220N, and M224V substitutions in human PRMT1V2 (isoform 1) (W197L, Y202N, M206V in rat PRMT1V1). Using a combination of site-directed mutagenesis, analytical ultracentrifugation, native PAGE, and activity assays, we found that these conservative substitutions surprisingly disrupt oligomer formation and substantially impair both S-adenosyl-L-methionine (AdoMet) binding and methyltransferase activity. Molecular dynamics simulations suggest that these substitutions introduce novel interactions within the dimerization arm that lock it in a conformation not conducive to dimer formation. These findings provide a clear, if putative, rationale for the contribution of these mutations to impaired arginine methylation in cells and corresponding health consequences.

Protein arginine methylation is a set of widespread posttranslational modifications that have been reported on a large number of both histone and nonhistone substrates (1, 2, 3, 4, 5, 6, 7, 8, 9, 10). In humans, arginine methylation is catalyzed by the nine S-adenosyl-L-methionine (AdoMet)-dependent members of the protein arginine methyltransferase (PRMT) family. Type I PRMTs form monomethylarginine (MMA) and asymmetric dimethylarginine (ADMA), Type II PRMTs form MMA and symmetric dimethylarginine (SDMA), and Type III PRMTs only form MMA (9, 10, 11, 12). The members of the PRMT family can work together or individually to help control a myriad of cellular processes including transcription, DNA repair, RNA splicing, and apoptosis by alterations to protein–protein or protein–nucleic acid interactions that result from arginine methylation of targeted proteins (9, 10, 11, 12). In recent years, the role of the PRMTs in the pathogenesis of several diseases, including cancer, has become increasingly appreciated (8, 13, 14, 15). While substantial progress in understanding the roles of the PRMTs in physiology and disease has been achieved in the last several years, the catalog of the consequences of arginine methylation is far from complete, and many aspects of the fundamental biochemistry of the PRMTs are still not fully understood.

PRMT1 is responsible for the majority of arginine methylation in mammalian cells (9, 16). This enzyme targets numerous proteins involved in epigenetic and transcriptional regulation such as histone H4 (17, 18), the estrogen receptor (19, 20), and the progesterone receptor (21). Upregulation of PRMT1 is observed in many cancer types and often correlates with cancer grade and poor patient prognosis (14, 22, 23, 24, 25, 26, 27, 28), and there are a growing number of studies suggesting that PRMT1 is an important regulator in many pathways that are dysregulated in cancers (14, 26, 29, 30, 31, 32, 33, 34, 35, 36). While it is clear that PRMT overexpression is deleterious in many diseases, it is also clear that maintaining some level of arginine methylation is critical for cellular and organismal health. This is evinced by the ubiquity of PRMT genes in eukaryotic organisms and the ubiquitous expression of PRMTs in different tissues (13, 14). In mice, whole organism knockout of the major PRMTs is lethal either during embryonic development or within a few moments after birth (37, 38, 39). In the context of cancer, one recent study showed that in non-*MYCN* amplified neuroblastoma, low PRMT1 expression was associated with decreased rates of cancer-free survival (40). Additionally, there are other examples of cancers in which decreased PRMT1 expression or PRMT1 knockdown is associated with worse outcomes (41, 42, 43). These considerations suggest that both increased and decreased arginine methylation activity has the potential to disrupt cellular homeostasis in various ways in different contexts.

Previous enzymological work on several PRMTs, including PRMT1, indicates that dimer formation is essential for activity (44, 45, 46, 47, 48, 49). The dimer is formed through interactions between a dimerization arm on one monomer and surfaces on a Rossman Fold on another monomer (44, 45, 46). Although a detailed mechanistic understanding of the requirement for dimerization has not been achieved, several studies have shown that perturbations to the dimer interface can impair PRMT1 methyltransferase activity (44, 45, 47, 48). As a way of linking the fundamental requirement for dimerization with biological consequences, we sought to identify if there were any mutations to the PRMT1 dimerization arm that might disrupt activity in characterized cancer cells. We searched the Catalogue of Somatic Mutations in Cancer (COSMIC) database (50) and identified three mutations at well-conserved sites in the human PRMT1 dimer arm (Fig. 1). Data from accelerated molecular dynamics (aMD) simulations indicated that these mutations were likely to disrupt activity. Using site-directed mutagenesis, analytical ultracentrifugation (AUC), native PAGE, and activity assays, we confirmed that oligomerization, AdoMet binding, and activity are all disrupted by all the mutations. Further investigation of the aMD trajectories indicated that the mutations likely stabilize the dimerization arm in a conformation incompatible with dimer formation.

**Figure 1:**
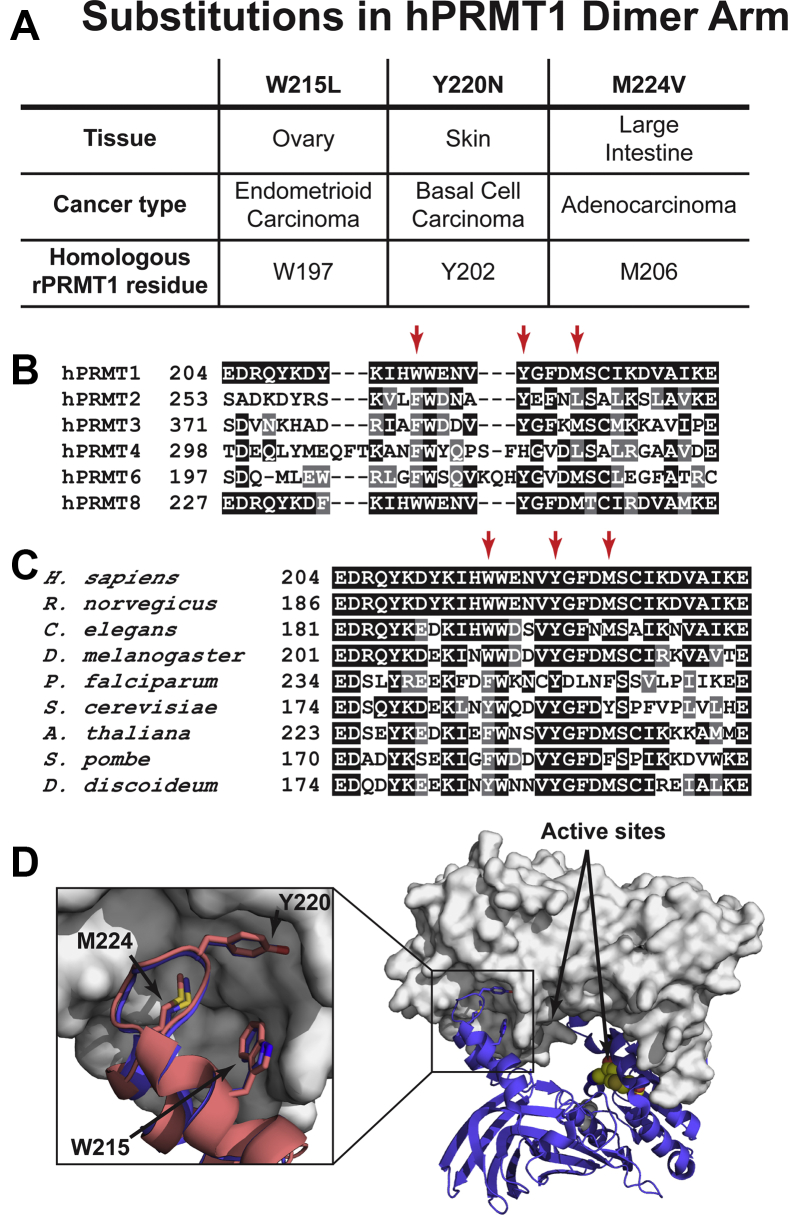
**PRMT1 dimerization arm residues mutated in human cancers with their sequence and structural conservation.***A*, mutated hPRMT1 dimerization arm residues with associated tissue and cancer types, and the homologous residues in rPRMT1. *B*, sequence alignment of the dimerization arm in human type I PRMTs. The residues mutated in hPRMT1 are indicated with *arrows*. *C*, sequence alignment of the dimerization arm in PRMT1 from several organisms. The residues mutated in hPRMT1 are indicated with *arrows*. *D*, structure alignment of a monomeric unit from the dimeric rPRMT1 crystal structure (PDB ID: 1OR8) to a dimeric unit from the hPRMT1 crystal structure (PDB ID: 6NT2). One of the subunits of the hPRMT1 dimer is shown as a surface with the other subunit shown as a *blue cartoon*. The AdoHcy molecule from the aligned rPRMT1 crystal structure is shown as *orange spheres* (AdoHcy/AdoMet is not present in the hPRMT1 structure). The subunit from rPRMT1 is shown as a *red cartoon* in the enlarged section. The positions of the two active sites and each mutated residue are indicated.

## Results

### Three mutations occur at well-conserved sites in the PRMT1 dimerization arm

Since PRMT1 dimerization is essential for activity, and aberrant arginine methylation seems to be significant in cancer pathology, we searched the COSMIC database for mutations in the PRMT1 dimerization arm. We found three mutations resulting in a change to the protein sequence, with one resulting in a W215L substitution, one in a Y220N substitution, and one in an M224V substitution (Fig. 1*A*). As a first step in determining if these mutations might be functionally relevant, we performed a sequence alignment of all type I PRMT paralogs in humans and an alignment of PRMT1 homologs across several species (Fig. 1, *B* and *C*). A Consurf (51, 52, 53, 54, 55) analysis of PRMT1 indicated that residues in the dimerization arm are not generally conserved as strictly as residues in other regions. Additionally, structures of available PRMTs indicate that the dimerization arm varies considerably in length and in the angle at which it protrudes from the core. However, we found relatively good conservation of W215, Y220, and M224 compared with the rest of the dimerization arm (Fig. 1, *B* and *C*). We hypothesized that these residues form important interactions that mediate dimer formation and that their mutations would disrupt both oligomerization and activity of PRMT1. We tested this hypothesis using *R. norvegicus* PRMT1 (rPRMT1), which differs in protein sequence from the comparable human splice variant by a single residue; Y179 in the *H. sapiens* PRMT1 (hPRMT1) Rossman Fold is ∼30 Å distal to the dimer interface and is a histidine in rPRMT1. The conformation of the dimerization arm in rPRMT1 and hPRMT1 crystal structures (Fig. 1*D*) shows an RMSD of 0.5 Å when the two arms are aligned, and previous work has shown that the methyltransferase rates measured from hPRMT1 and rPRMT1 are similar for most substrates (56). Finally, we have previously studied rPRMT1 using both experimental and computational methods and have accumulated a strong foundation for comparison studies (7, 11, 57, 58, 59, 60, 61, 62, 63, 64, 65, 66).

### NAC analysis predicts low probability of catalysis with all cancer mutations

We have previously had success modeling active-site changes in PRMT1 and PRMT7 by analyzing molecular dynamics (MD) trajectories. In particular we have used quantum mechanics (QM) on AdoMet and the substrate arginine to calculate the transition state barriers for ADMA *versus* SDMA formation to understand product specificity in a PRMT1 variant with a remodeled active site (58), and we used both MD trajectories and mixed quantum mechanics and molecular mechanics (QM/MM) coupled with MD sampling to investigate product formation in a suite of PRMT1 (57) and TbPRMT7 (67, 68) variants to compare probabilities for monomethylation and dimethylation. These studies either considered the reactants (AdoMet and the substrate arginine) in isolation, used the WT enzyme, or used variants with mutations directly in the active site. As part of an ongoing effort to develop and refine *in silico* analyses sensitive to dynamics occurring across the entire catalytic core, we expanded this approach to the three dimerization arm mutations outside of the active site.

**Figure 2:**
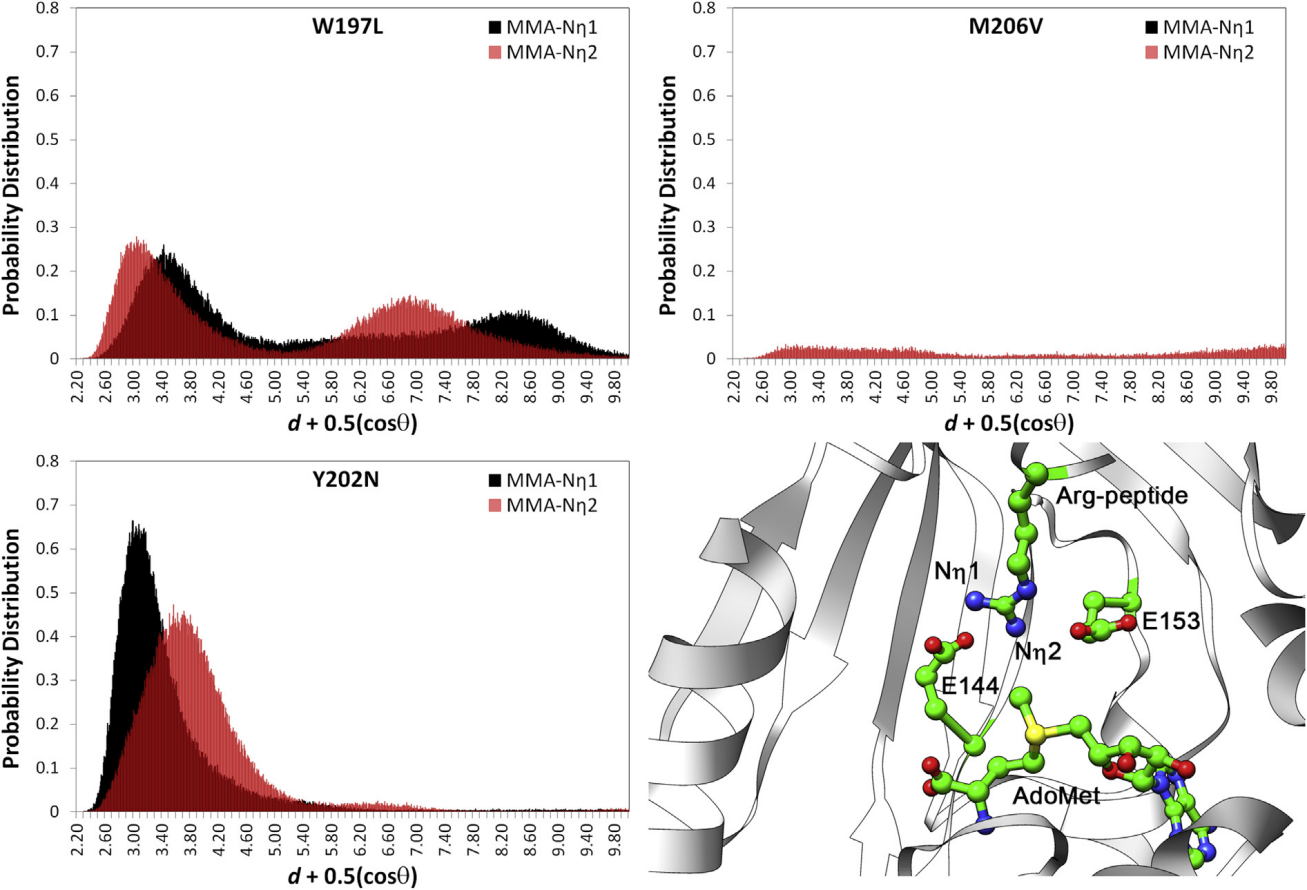
**Probability of sampling near-attack conformers (NACs) in the monomers of three individual naturally occurring mutations (W197L, Y202N, and M206V) of PRMT1 that lead to the formation of the MMA product.** The preferred orientations by the substrate over time were evaluated by comparing the distributions of *d* + 0.5(cosθ) over the trajectory, where *d* is the attack distance between the nitrogen atom in arginine (N_η1_ or N_η2_) and the methyl carbon in AdoMet, and θ is the respective attack angle (N^…^CH_3_^…^S). The *bottom right panel* shows the N_η1_ and N_η2_ of the substrate arginine residue in the active site. The enzyme is shown as a *white cartoon*, with E144, E153, the peptide substrate, and the AdoMet molecule highlighted in *green*.

The three individual cancer mutations of PRMT1, *i.e.*, W197L, Y202N, and M206V, were explored computationally by investigating the thermodynamic stabilization of near attack conformations (NACs) that resemble the transition state. NACs are defined as possessing reacting atom distances within 3.2 Å and an approach angle of ±15° of the ideal 180° bonding angle in the S_N_2 transition state (69). In the current study, the geometric orientations of the substrates within the three variant PRMT1 monomers were examined using 1000 ns aMD simulations to determine the probability of sampling near the S_N_2 transition states that lead to the formation of the MMA product. The preferred orientations of the substrates over time were evaluated by comparing the trajectory distributions of *d* + 0.5(cosθ), where *d* is the attack distance between the nitrogen atom in arginine (N_η1_ or N_η2_) (Fig. 2) and the methyl carbon in AdoMet, and θ is the respective attack angle (N^…^CH_3_^…^S). The most ideal S_N_2 attack angle of θ = π radians (180°) will favorably scale down the final *d* value by −0.5. Therefore, the scaling rewards a productive S_N_2 angle between 90 and 180° and penalizes angles <90°. Substantial differences exist between the three PRMT1 cancer-based mutations in terms of the probability of forming a NAC to yield MMA (Fig. 2). For example, the Y202N PRMT1 variant was predicted to be the most likely of the three mutants to be active with a preference for S_N_2 attack by the N_η1_ atom of the arginine substrate with a tall narrow peak centered around 3.00 Å at a height of 0.66 (as a reference, previous studies with WT PRMT1 showed peak heights between 1.0 and 1.2 (57, 67). However, the M206V variant found the exact opposite trend, with virtually no formation of NAC conformers even at distances up to 10 Å. The W197L variant featured NAC formation probabilities that were somewhere in between the other two cancer variants with a peak centered around 3.14 Å, but with a much lower probability distribution of 0.27 for the more favorable N_η2_ atom attack. The NAC analyses suggest that all PRMT1 single mutants would have impaired, if any, activity.

### Methyltransferase activity of PRMT1 is altered in the variant constructs

**Figure 3:**
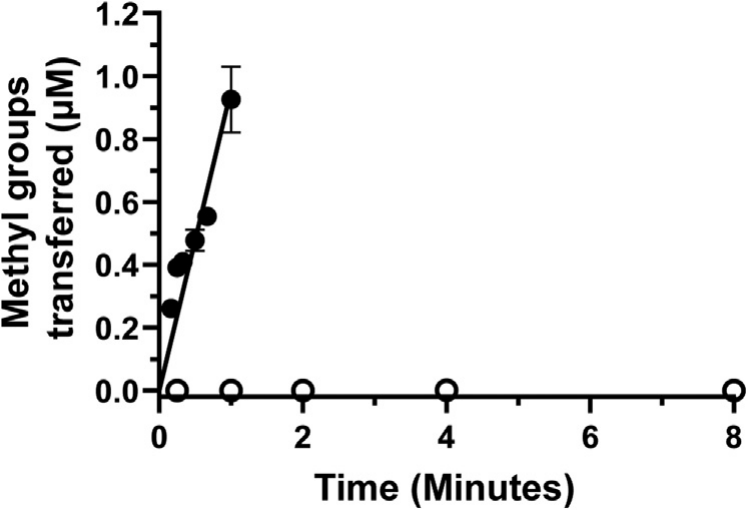
**Activity of WT and W197L/Y202N/M206V rPRMT1.** Activity was assessed with 200 μM R3 peptide substrate, 2 μM AdoMet, and 750 nM WT (*black circles*) or triple mutant (*open circles*).

In order to determine whether any of the three mutations identified in the COSMIC database were functionally relevant *in vitro*, we first characterized the W197L/Y202N/M206V rPRMT1 triple mutant. Given that our NAC analyses predicted little to no activity from the variant constructs, we hypothesized that a construct harboring all three mutations would be inactive. We tested the activity of WT and the triple mutant using a discontinuous assay that employs radiolabeled AdoMet as a tracer (62, 66). Typically, this assay is conducted at a low enzyme concentration of 100 nM to ensure a sustained steady-state. However, because we anticipated that the triple mutant construct would show impaired activity, we increased the enzyme concentration to 750 nM. While observing robust activity from the wild-type construct, we were unable to observe any methyltransferase activity from the triple mutant construct under these conditions (Fig. 3). Even at enzyme concentrations as high as 3 μM, we were unable to detect any methyltransferase activity from the triple mutant construct (data not shown). We conclude that the W197L/Y202N/M206V variant of rPRMT1 is inactive.

**Figure 4:**
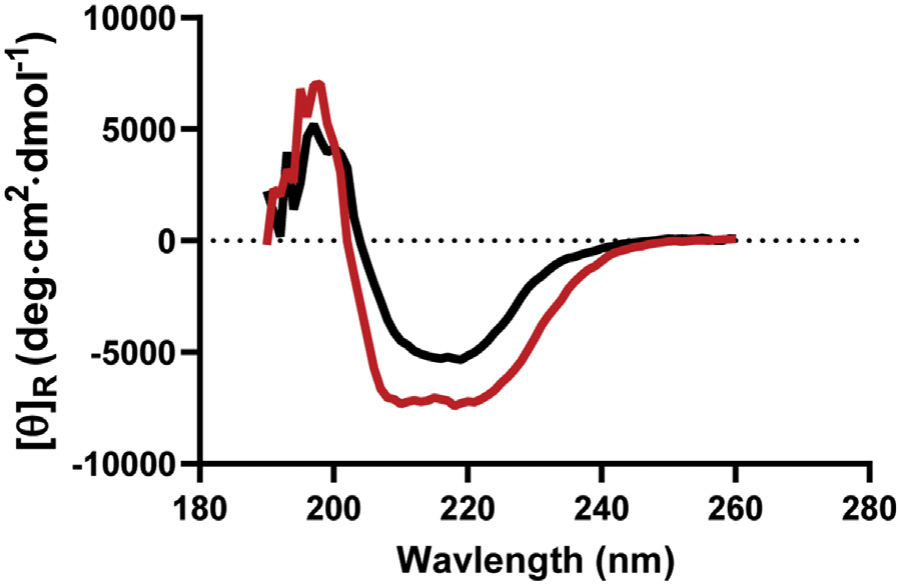
**CD spectra for WT (*black*) and W197L/Y202N/M206V PRMT1 (*red*)**.

It is possible that the observed absence of activity in the W197L/Y202N/M206V construct results from an overall destabilization of the enzyme, rather than through perturbation of the dimer arm. We used CD spectroscopy to assess secondary structure content of the wild-type and triple mutant constructs. Interestingly, the CD spectra for the two constructs vary, with an overall *increase* in signal in the triple mutant, particularly in the regions near 208 and 220 nm (Fig. 4 black *versus* red traces). This increase in signal for the mutant in comparison to WT indicates that the mutations do not cause gross unfolding, but instead suggests an increase in alpha helical content in the triple mutant. Interestingly, a general increase in alpha helical content of the PRMT1 N-terminus was observed in the monomer compared with the dimer in a previous computational study (48), suggesting that the triple mutant may be monomeric.

**Figure 5:**
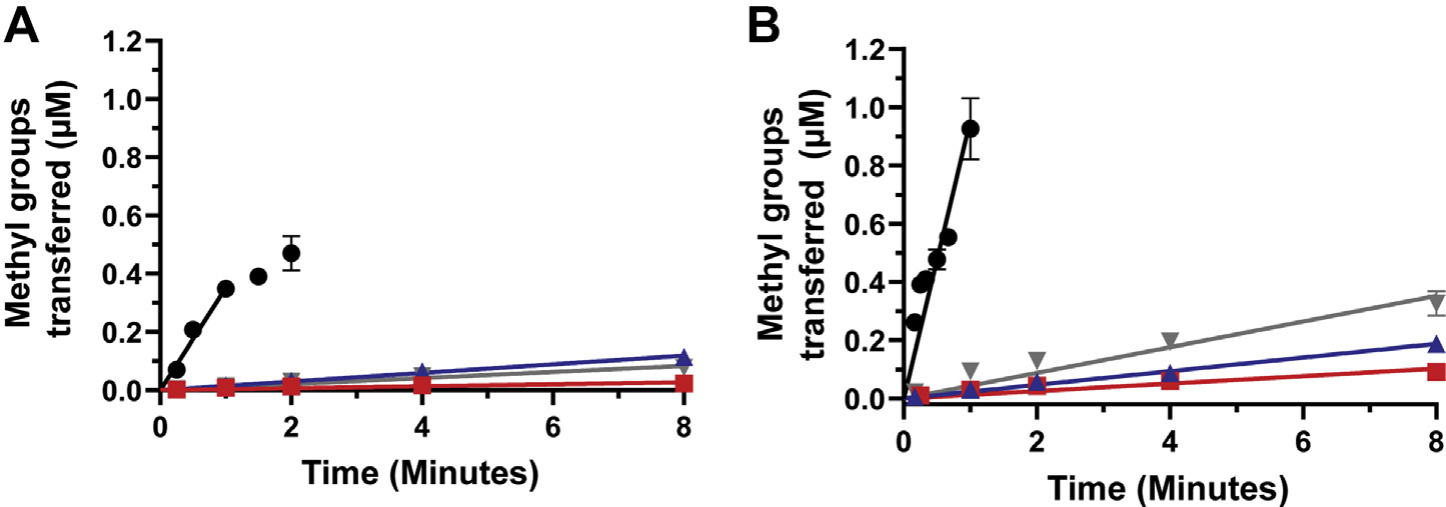
**Activity of WT and dimer arm rPRMT1 single mutants.** Activity was assessed with 200 μM R3 peptide substrate, 2 μM AdoMet, and varying enzyme concentrations. *A*, enzyme activity assessed at 200 nM with WT PRMT1 (*black circles*), W197L PRMT1 (*red squares*), Y202N PRMT1 (*blue triangles*), and M206V PRMT1 (*gray inverted triangles*). *B*, enzyme activity assessed at 750 nM with WT PRMT1 (*black circles*), W197L PRMT1 (*red squares*), Y202N PRMT1 (*blue triangles*), and M206V PRMT1 (*gray inverted triangles*).

Each mutation in the triple mutant construct occurs individually in different cell lines. In order to test the contribution of each mutation to the loss of activity observed in the triple mutant, we generated W197L, Y202N, and M206V single mutant constructs. Initial assays at an enzyme concentration of 100 nM yielded a signal from each mutant that was higher than background, but not high enough to confidently conclude that the single mutant constructs were active. Thus, we increased the enzyme concentration in subsequent assays to 200 nM and 750 nM. Once again, the wild-type construct showed robust activity, quickly consuming available AdoMet, which is particularly apparent at longer timepoints (not shown). While this loss of the steady state in the wild-type assay precludes quantitative comparisons, it is clear that all three single mutant constructs show low and roughly comparable levels of activity compared with wild type (Fig. 5, *A* and *B*).

### Oligomerization of PRMT1 is altered in the variant constructs

**Figure 6:**
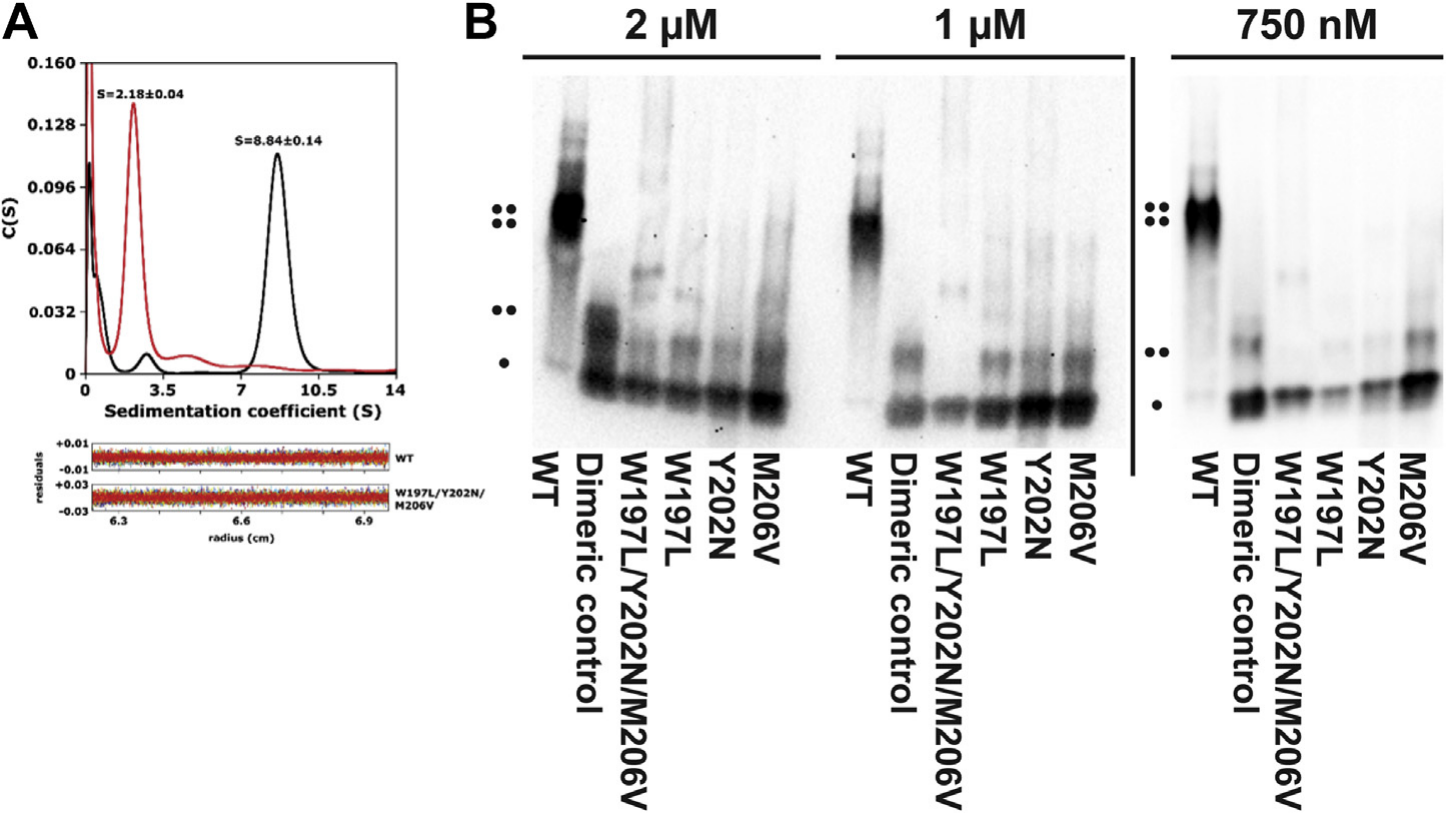
**Oligomeric status of the PRMT1 variants.** In (*A*) the oligomeric state of WT PRMT1 (*black*) and the W197L/Y202N/M206V variant of PRMT1 (*red*) were assessed using analytical ultracentrifugation. The major peaks for WT PRMT1 and W197L/Y202N/M206V rPRMT1 have sedimentation coefficients of 8.84 ± 0.14 and 2.18 ± 0.04 respectively. These peaks correspond to masses of 198 kDa and 40.2 kDa, which is consistent with a tetramer for WT (monomer mass = 43.5 kDa) and a monomer for the W197L/Y202N/M206V variant (monomer mass = 43.3 kDa). Residuals are shown below the plot. The protein concentration for both samples was 3 μM. In (*B*), the oligomeric state of WT PRMT1, the W197L/Y202N/M206V variant of PRMT1, and each of the single mutant constructs were assessed using 4 to 20% native PAGE with western detection. PRMT1 concentrations at 2 μM, 1 μM, and 750 nM are shown in (*B*). Images separated by the *vertical line* are different membranes. The *dots* to the side of the blot indicate tetramer, dimer, and monomer species.

Given that dimerization is important for catalysis (45, 47, 48), and that each of the dimer arm mutations disrupted activity, we wondered if they also disrupted normal dimerization. We characterized the native molecular species present in both WT and W197L/Y202N/M206V rPRMT1 samples using AUC (Fig. 6*A*). We found that at the lower limit of detection (3 μM), the triple mutant was predominantly monomeric with a small amount of dimer, and that WT was predominantly tetrameric with a small amount of monomer (Fig. 6*A*). These measurements were taken at a concentration much higher than the range in which activity is routinely measured in biochemical studies. Additionally, it also seems likely that physiological concentrations of PRMT1 are much lower than 3 μM. Thus, in order to determine the oligomeric state of each construct at lower concentrations, we used native PAGE with downstream western blotting. In order to confidently assign the bands observed in native PAGE, we standardized migration distances for the tetramer using WT PRMT1 and for the dimer using a previously characterized tetramerization-deficient PRMT1 mutant (70). We detected almost no dimer in the triple mutant construct by native PAGE at 750 nM and 1 μM, with a small amount of dimer appearing at 2 μM (Fig. 6*B*). These results confirm that the three mutations together significantly impair dimerization and higher-order oligomer formation.

Each of the single mutant constructs also showed impaired oligomerization at all concentrations tested, although a small fraction of dimer (5–20%) can be seen, particularly at the higher concentrations of 1 and 2 μM. These results show that even a single mutation in the dimer interface at W197, Y202, or M206 in rPRMT1 is sufficient to impair normal oligomerization.

### Dimer arm mutations impair AdoMet binding in PRMT1

**Figure 7:**
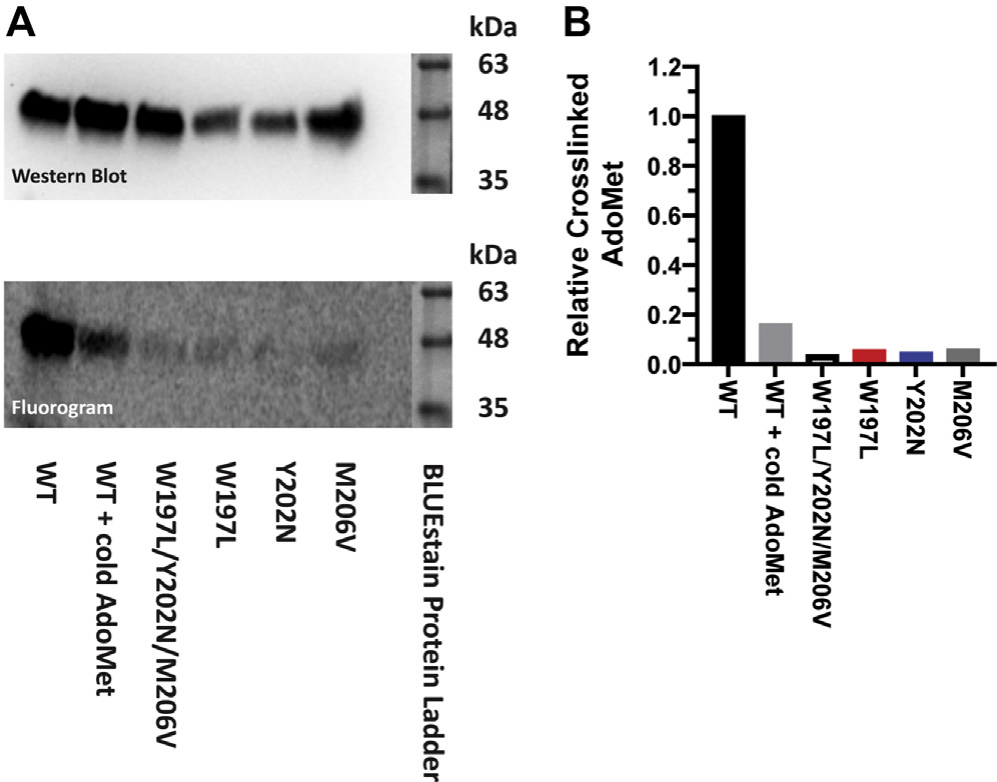
**Mutations in the PRMT1 dimer arm impair AdoMet binding.** 750 nM protein samples were UV cross-linked to tritiated AdoMet, then blotted onto a PVDF membrane. The membrane was used to expose a phosphor screen, then analyzed by western blotting. Unlabeled AdoMet was added to a duplicate WT reaction to compete with tritiated AdoMet and ensure that the cross-linked AdoMet was specifically bound. Molecular weight estimates for the western blot were obtained using a colored ladder which was imaged during the same imaging session as the western blot. Molecular weight estimates for the phosphor image were based on estimates from the western blot, with the images being matched by overlaying smearing patterns and other irregularities visible in both the western blot and phosphor image. Ladder bands in both images are overlaid from a separate image capture of the colored ladder bands. *A*, top: α-His blot of UV/AdoMet cross-linked PRMT1. *Bottom*: Fluorogram of the blot shown in the *top panel*. *B*, relative cross-linked AdoMet determined as the cross-linking signal intensity divided by the corresponding western blot signal intensity.

Several studies have indicated that PRMT1 constructs deficient in dimerization are also deficient in AdoMet binding (44, 45, 47, 48). It was previously suggested that dimerization may aid in conformational changes that expose the AdoMet-binding pocket (48) making AdoMet binding more likely. Thus, we sought to determine if AdoMet binding was perturbed in the mutant constructs. This was done using a radiolabeled AdoMet cross-linking assay (16, 44, 71) (Fig. 7). We observed that the WT construct is capable of binding tritiated AdoMet (lane 1) and that unlabeled AdoMet can compete with tritiated AdoMet for binding (lane 2), indicating that the cross-linked AdoMet is specifically bound. The variant constructs showed very little AdoMet binding compared with wild type. This indicates that AdoMet binding is severely impaired in the mutant constructs.

### Cluster analysis detects predominant structures for mutant monomeric species

In one respect, the fact that a single mutation in the dimer arm can nearly abolish dimer formation seems surprising, especially in cases where the new residue is smaller than the WT residue in a large dimerization interface. However, our experimental results show that the W197L, Y202N, and M206V substitutions each substantially impair PRMT1 function. Each of these mutations is relatively conservative, and from a first-principles perspective it is difficult to rationalize how these subtle alterations affect PRMT1 oligomerization and function so drastically. Using the PDBePISA service (72, 73), we determined that the dimer interface involves interactions between 78 residues (39 per monomer) with a total buried surface area of ∼3000 Å^2^ (∼1500 Å^2^ from each monomer). Each of the single mutations made in this study alters two residues (one per monomer). Using PyMol (74) we calculated that W197 accounts for a buried surface area of only 123 Å^2^ per dimer (4% of the total buried surface), Y202 accounts for 343 Å^2^ (11% of the buried surface), and M206 accounts for 130 Å^2^ (4% of the buried surface). Each residue accounts for a fairly small fraction of the interface and would not be expected to make a substantial contribution to the total binding energy of dimerization. While Y202 contributes to two inter-subunit hydrogen bonds (one per monomer), there are a total of ten hydrogen bonds and two salt bridges at the interface (72, 73) and loss of two hydrogen bonds would not be expected to substantially alter the total binding energy of dimerization. Thus, it seems unlikely that the perturbed dimerization observed in this study results from a substantial loss of interactions occurring at the dimer interface. Furthermore, the W197L, Y202N, and M206V substitutions each introduce residues that are a similar size or smaller, so it does not seem likely that the perturbed dimerization arises from steric effects.

**Figure 8:**
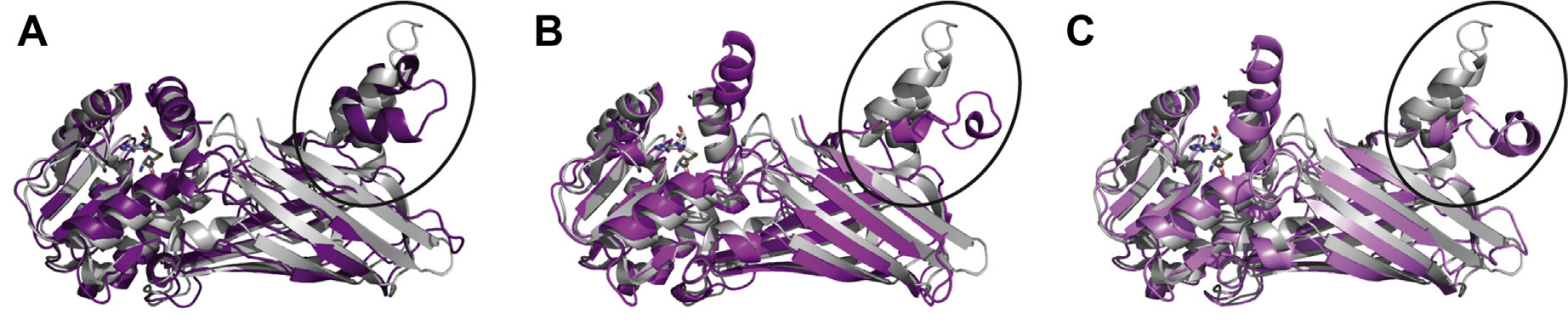
**Conformation of WT rPRMT1 monomer simulations (*shades of purple*) compared with the rPRMT1 crystal structure (*gray*, PDB ID:****1OR8****).***A*, the predominant WT monomer structure (26.2%). *B*, the second most common WT monomer structure (23.4%). *C*, the third most common WT monomer structure (12.6%).

In an effort to better understand why the single site mutations in the dimer arm impaired dimerization so significantly, clustering analysis was performed on the 1000 ns aMD trajectories in order to identify the most dominant structures for each PRMT1 system. The simulations indicated that the WT monomer had three major clusters that encompassed similar amounts of the trajectory time (26.2%, 23.4%, and 12.6%) (Table 1). In each cluster the dimerization arm is in a slightly different conformation (Fig. 8) suggesting that this region of the WT monomer may be flexible, but in each case the hydrophobic residues that comprise the dimerization interface fold away from where the dimer interface would be and instead pack against one another. The arm as a whole is shifted away from the position observed in the dimeric crystal structure and instead packs against the beta barrel domain. This is consistent with a previous study investigating the relationship between dimerization and AdoMet binding (48). In that study, similar destabilization and conformational rearrangements were seen in an MD trajectory with monomeric WT PRMT1. There the authors calculated a rough energy landscape for the dimerization arm in the PRMT1 monomer and identified two energy wells separated by a low barrier, each corresponding to conformations in which the dimerization arm packed against the beta barrel domain. This suggests that the dimerization arm is flexible in the monomer, and that the extended dimerization arm conformation observed in the crystal structure is stabilized by contacts formed upon dimerization. In contrast to these observations of WT PRMT1, in our study each of the mutant monomers displayed a single dominant structure throughout the aMD trajectory (Table 1).

**Table 1:** Prevalence (%)^a^ of the top three clusters during aMD simulation

WT			W197L/Y202N/M206V			W197L			Y202N			M206V		
26.2	23.4	12.6	84.7	6.0	3.5	86.5	9.2	2.1	85.0	4.8	3.9	61.9	24.6	6.5

a Percentage of time each construct spent in each of the top three conformation clusters throughout the aMD trajectory.

**Figure 9:**
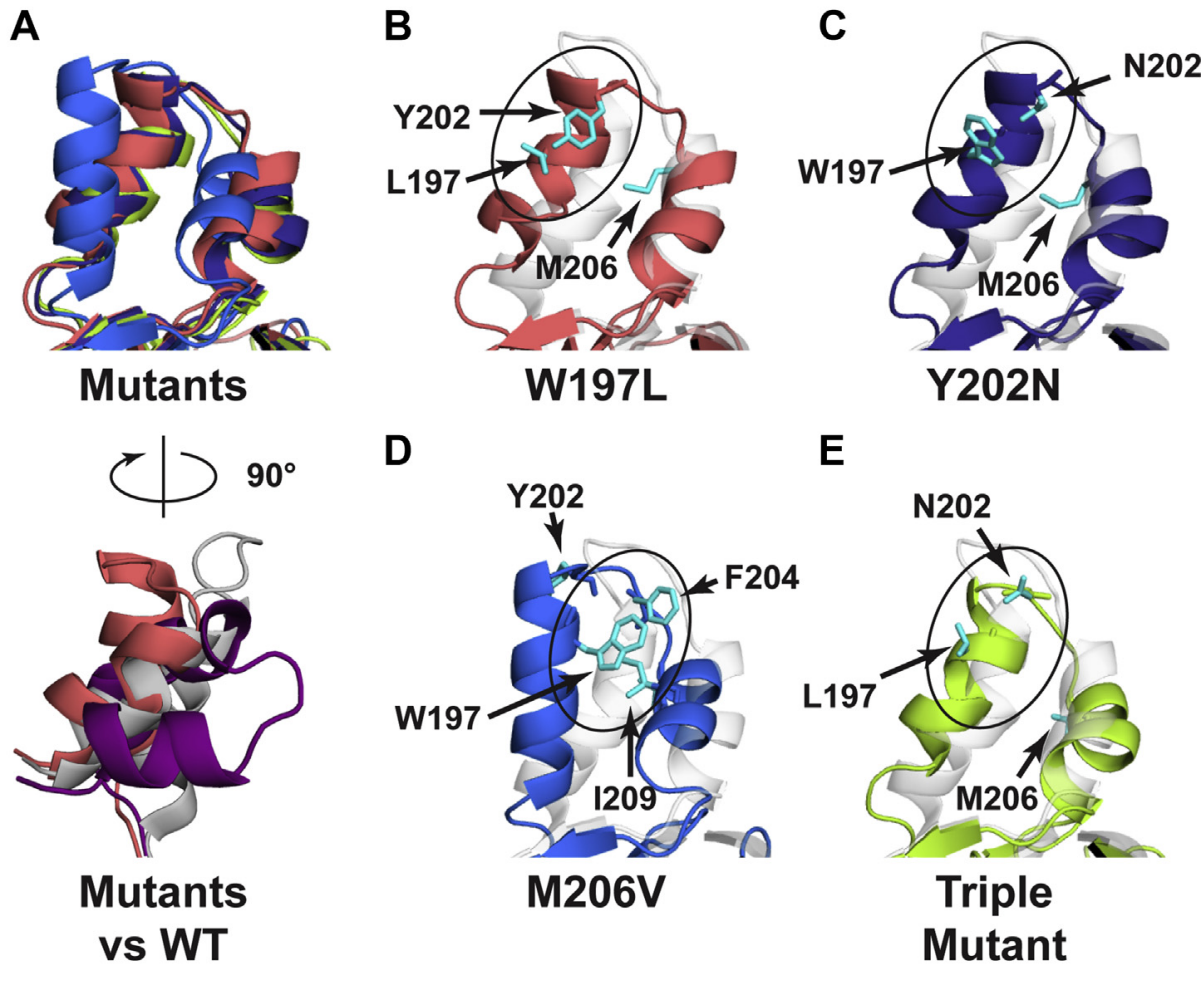
**Alignment of the rPRMT1 dimerization arm and new interactions created in W197L (*salmon pink*), Y202N (*dark blue*), M206V (*light blue*), and W197L/Y202N/M206V (*light green*) monomers, with comparison to the dimeric wild-type crystal structure (*light gray*) and the dominant simulated WT monomer (purple).** In panels *B*–*E,* highlighted residues are shown in *cyan*. The crystal structure is shown as a ghosted cartoon. The dominant clusters for W197L, Y202N, and the triple mutant are each present about 85% of the time. The dominant structure for M206V is present 61.9% of the time. *A*, *top*: Aligned dimerization arm from each mutant structure. *Bottom*: The W197L dimerization arm (*salmon pink*) is taken as representative of the conformation of each mutant and compared with the WT crystal structure (*light gray*), and to the predominant WT simulation structure (*purple*). The view in the *bottom panel* is rotated ∼90° relative to the *top panel* and is taken from the same viewing plane shown in Figure 8. *B*, in W197L, the face of Y202 packs against L197 and interacts through van der Waals contacts. While these van der Waals contacts might be expected to be relatively weak, the interaction may also be stabilized by the hydrophobic effect. *C*, in Y202N, N202 hydrogen bonds with the backbone of W197 and the face of W197 appears to contact the amide nitrogen of N202. *D*, in M206V, Y202 and V206 are both oriented outward (V206 not visible in structure shown). W197 appears to contact F204 and I209. *E*, in W197L/Y202N/M206V, the backbone and side chain of N202 both hydrogen bond with the backbone carbonyl of L197.

In order to understand how the mutations identified in this study might disrupt dimerization, we examined the most dominant cluster structures of each monomeric mutant. We were surprised to find that the predominant cluster for each mutant adopted a similar fold distinct from each of the compact conformations observed in the WT monomer and also from the extended conformation observed in the WT crystal structure (Fig. 9*A*). Interestingly, the W197L/Y202N/M206V, W197L, and Y202N variants each adopt a nearly superimposable conformation. This conformation may be stabilized by interactions between the residues at positions 197 and 202 observed in each of these variants but not observed in the WT structure (Fig. 9, *A*–*C* and *E*). In the M206V cluster, new interactions are also observed in the dimerization arm, but not between residues 197 and 202. Instead, the loop harboring residue 202 is oriented outward away from the rest of the arm, and W197 packs against F204 and I209. The valine introduced at position 206 is not involved in this interaction, and it points back toward the solvent-exposed face of the arm. It is possible that one role for M206 in the WT enzyme is to block these interactions from occurring. While V206 is not directly involved in any new interactions in the dimerization arm, the M206V substitution may alter the conformational energy landscape to favor the unique conformation observed in the mutants. Overall results from the cluster analysis suggest that each dimer arm mutation similarly impairs dimerization through a mechanism in which the dimerization arm is stabilized in a conformation that is not conducive to dimer formation.

## Discussion

### W197L, Y202N, and M206V substitutions disrupt oligomerization and activity in PRMT1

The reactions catalyzed by PRMT1 are crucial for maintaining cellular health, and dysregulated PRMT1 contributes to the pathology of several cancers (14, 22, 23, 24, 25, 26, 27, 28). In this study we identified three mechanistically relevant dimer arm mutations by screening mutations that occur in human cancers. These mutations occur at well-conserved sites, and our work indicates that they each disrupt PRMT1 dimerization, AdoMet binding, and activity. Our computational results suggest that this likely occurs through a novel mechanism in which the dimerization arm becomes locked in an unfavorable conformation by new interactions within the dimerization arm induced by each mutation.

**Figure 10:**
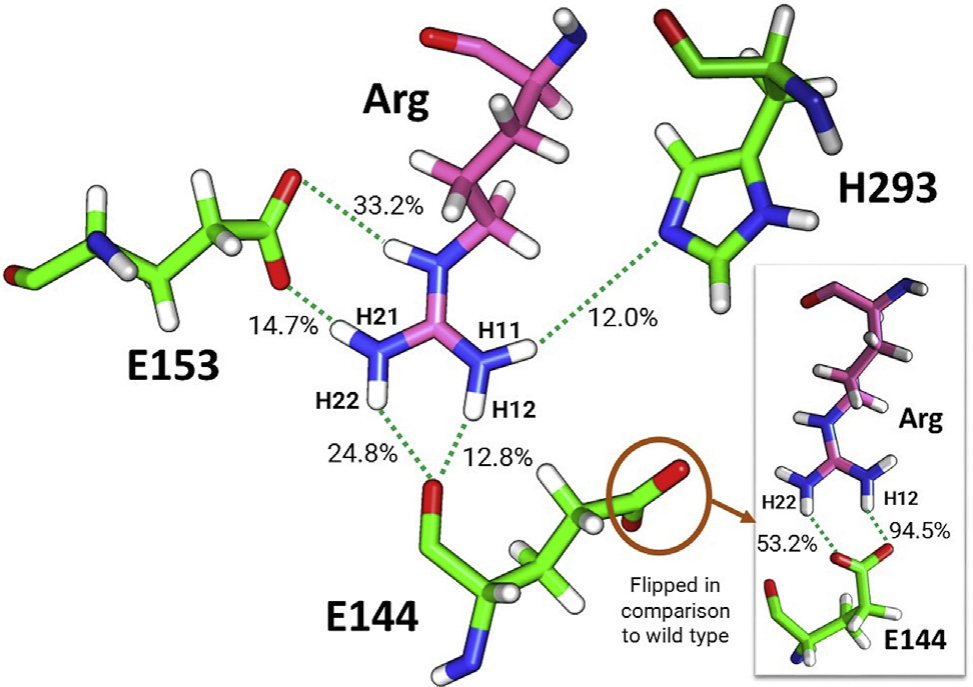
**The representative active site orientation and percent hydrogen bonding computed over the entire 1000 ns aMD trajectory of the most dominant Y202N PRMT1 cluster.** The side-chain carboxylate group of E144 is flipped away from the peptide arginine in stark contrast to WT PRMT1 (shown in the *insert*).

In this study, the NAC analysis unambiguously suggested that two of the rPRMT1 constructs with mutations distal to the active site (W197L and M206V) would have decreased activity, but the results were less clear for the Y202N variant. However, the NAC analysis applied here only considers the distance and angle between the methyl carbon and the methylated nitrogen, ignoring factors that contribute to the nucleophilic activation of the nitrogen. In our earlier studies a strong hydrogen bond was observed to form between the substrate arginine and wild-type PRMT1 active site residues E144, E153, H293, and D51 (57). The proper orientation of the substrate arginine was found to be very important for S_N_2 methyl attack (67). These active-site interactions may provide a diagnostic indicator to help explain why Y202N activity was impaired as strongly as it was, even though the NAC analysis was for more favorable for Y202N than the other variants. Inspection of the most dominant Y202N cluster structure from the aMD simulations found that while the substrate arginine appeared to be anchored in the pocket by the H293 and E153 active site residues, the E144 active site base was flipped away from the reacting arginine (Fig. 10). Thus, despite the computed increased probability of NAC formation for Y202N PRMT1 relative to the M206V and W197L variants, an improper active site base orientation in Y202N likely hinders reactivity. This hypothesis was further examined by computing the hydrogen bonding percentage between the active site residues and Arg over the entire 1000 ns aMD trajectory by using the cpptraj module of Amber16 with a cutoff distance and angle of 3 Å and 135°, respectively (57, 58, 75, 76). When compared to with WT PRMT1, the Y202N variant found substantially reduced hydrogen bonding present between the peptide Arg and all of the active site residues, *i.e.*, E153, E144, and H293 (Table 2). This suggests a very loosely anchored Arg peptide in the Y202N PRMT1-binding pocket. For example, previous WT PRMT1 simulations reported 94.5% and 53.2% hydrogen bonding present between the side-chain carboxylate oxygen atoms of E144 and the Arg guanidino group (57), whereas in Y202N these values dropped substantially to 5.7% and 3.4%, respectively (Table 2). Instead, the backbone carbonyl oxygen atom of E144 interacted with the Arg guanidino group through computed hydrogen bonding of 24.8% and 12.8% (Fig. 10). In addition, the hydrogen bonding percent between Arg and the carboxylate oxygen atoms of E153 dropped to 33.2% and 14.7% in Y202N as compared to with 92.2% and 62.6% in the wild type, respectively. Finally, the interaction between H293 and Arg was reduced to 12.0% in Y202N compared to with 60.4% in WT PRMT1. These considerations are consistent with the loss of activity we observe in the Y202N variant. Our results show that a computational approach that combines both NAC analysis and active site hydrogen bonding architecture tracks well with experimental measures of activity, even when the mutations under study are distal to the active site.

**Table 2:** Computed percent hydrogen bonding between the Y202N PRMT1 active site residues and the guanidino group of the bound Arg peptide over the 1000 ns aMD trajectory

HB acceptor	HB Donor	WT [57]	Y202N
Glu144@OE(1+2)	ARG@HH12	94.5%	5.7%
Glu144@OE(1+2)	ARG@HH22	53.2%	3.4%
Glu144@O	ARG@HH(21+22)	N/A	24.8%
Glu144@O	ARG@HH(11+12)	N/A	12.8%
Glu153@OE(1+2)	ARG@HE	92.2%	33.2%
Glu153@OE(1+2)	ARG@HH21	62.6%	14.7%
Glu153@OE(1+2)	ARG@HH(11+12)	N/A	26.7%
His293@NE2	ARG@HH11	60.4%	12.0%

A comparison is made to previously reported simulations of WT PRMT1.

Previous investigations have shown a positive correlation between AdoMet binding and oligomer formation (48, 77). One recent investigation suggested the existence of an allosteric pathway involving residues between the active site, the dimerization arm, and the dimer interface on the Rossman Fold. The authors suggested a mechanism in which AdoMet binding is communicated to the dimerization interfaces, stabilizing dimerization, which in turn helps to stabilize and regulate the active site (including stabilizing the N-terminal helix that covers the AdoMet-binding pocket) (48). Our findings that AdoMet binding is nearly abolished in the W197L, Y202N, M206V, and monomeric triple mutant constructs are consistent with this model.

Interestingly, allosteric PRMT3 inhibitors that occupy a pocket at the base of the dimerization arm have been identified (78, 79, 80, 81). Comparisons of crystal structures in the presence and absence of the inhibitors show very similar conformations in the dimerization arm; however, the presence of the inhibitor at the base of the arm may alter conformational dynamics involving the arm that may not be obvious in the crystalline state. Taken together, the collective results support the suggestion that conformational dynamics in the dimerization arm are critical for catalysis.

### Is PRMT1 a morpheein?

A morpheein is a protein in which lower oligomeric forms are capable of adopting distinct conformations, which give rise to structurally distinct higher-order oligomers with distinct functions (82, 83, 84). Some of the characteristics of morpheeins are hinted at in the results of this study. Our native PAGE blots showed that WT PRMT1 is predominantly tetrameric while each mutant showed a mixture of monomers and dimers (Fig. 6*B*). Close inspection of the membranes reveals several minor bands in the mutant constructs that migrate between the tetramer and the dimer. Some of these bands may represent alternative morpheein oligomers that become more prevalent in the mutant construct. Additionally, in our initial purification efforts with the mutant constructs, we observed large amounts of persistent contaminating nucleic acids. While characterizing this potential nucleic acid binding was not one of the goals of this study, we found that WT purifications with a high A260/280 ratio showed negligible differences in activity when compared with purifications with a low A260/280 ratio. Direct nucleic acid binding has been reported for TbPRMTs 1 and 7 (85), but it is unclear if this nucleic acid binding is related to a moonlighting function or if it has any physiological relevance. However, a moonlighting function has been reported for PRMT8, which is capable of phospholipase activity (86). While poor yields from our PRMT1 dimer arm mutant purifications preclude detailed kinetic and structural characterization, continued methodological improvements may change this in the future. These studies, and more detailed investigations of PRMTs as morpheeins (87) may reveal broader roles for the PRMTs than are currently known.

### PRMT mutations are continuing to be reported

New missense mutations (V219M, C226Y, and D229Y) have been reported in the hPRMT1 dimerization arm since we began work on this study. Additional missense mutations in the dimerization arm of other type I PRMTs include 6 missense mutations in hPRMT2, 8 in hPRMT3, 8 in hPRMT4, 10 in hPRMT6, and 18 in hPRMT8. In addition to these missense mutations, there are 6 silent mutations in hPRMT1, 5 in hPRMT2, 1 in hPRMT3, 4 in hPRMT4, 2 in hPRMT6, and 7 in hPRMT8. There are also nonsense mutations in the codons for PRMT1 W216, PRMT3 W383, and PRMT8 W238. It is probable that many of these missense mutations and all of the nonsense mutations have deleterious effects on PRMT activity. While these silent mutations may not be expected to significantly alter the function of the encoded protein, it is now well established that silent mutations can have a variety of deleterious consequences, which include alterations to rates of protein expression, changes to mRNA regulation, and altered protein folding (88, 89, 90, 91). Although available evidence suggests that PRMTs play an important role in many cancers, few studies have considered PRMT mutations as a contributor to pathology. However, it does not appear to be the case that PRMT1 mutations are generally rare in cancers, with 9.0% (1024/11,315) of samples in The Cancer Genome Atlas (TCGA) harboring PRMT1 mutations. For a rough comparison, 11.6% of TCGA samples have mutations to Ras, which is a well-known oncogene (92). Given the results reported here and the growing number of reported PRMT mutations with potential functional consequences, the lack of mutational studies may be an oversight.

## Experimental procedures

### Identification of cancer-associated mutations and sequence alignment of PRMT variants

In order to identify mutations within the PRMT1 dimerization arm, we search the COSMIC database (50) for PRMT1. We searched transcript ENST00000454376.6, ENST000003918151.8, and ENST00000532489.5 for mutations to the dimerization arm, which we defined as the helix-turn-helix motif that occurs between residues 204 and 234 in hPRMT1V2. For the sequence alignment to analyze sequence conservation in the dimerization arm, we used the Clustal Omega (93, 94) web server with the output format in Pearson/FASTA and all other settings set to the default parameters. Alignments were colored according to similarity using Boxshade, then manually truncated to the relevant regions. Sequences used for the alignments were as follows: human PRMT1 – Q99873, human PRMT2 – P55345, human PRMT3 – O60678, human PRMT4 - Q86X55, human PRMT6 - Q96LA8, human PRMT8 - Q9NR22, *R. norvegicus* PRMT1 – Q63009, *A. thaliana* PRMT1 – Q9SU94, *D. discoideum* PRMT1 – Q54EF2, *D. melanogaster* PRMT1 – Q9VGW7, *P. Falciparum* PRMT1 – Q8ILK1, *S. cerevisiae* PRMT1 – P38074, *S. pombe* PRMT1 – Q9URX7, *C. elegans* PRMT1 – Q9U2X0.

### Design, expression, and purification of PRMT1 variants

The wild-type rPRMT1 was coded by a pET28b vector coding for a His(6) tag with thrombin and TEV cleavage sites (59). The genes for rPRMT1 W197L/Y202N/M206V and each single mutant were designed based on the WT rPRMT1 sequence by changing the relevant codons from TGG to CTG (W197L), TAT to AAT (Y202N), and ATG to GTG (M206V). The genes were synthesized and then subcloned into a pET28 vector between the *Nco*I and *Bam*HI sites by General Biosystems.

Plasmids were transformed into BL21 DE3 *Escherichia coli* cells and grown overnight (12 h) on LB agar containing 35 μg/ml kanamycin at 37 °C then stored at 4 °C. Starter cultures were grown by inoculating a colony from the transformation plate into 50 ml of LB media containing 35 μg/ml kanamycin and shaking in a 250 ml baffled Erlenmeyer flask at 37 °C and 200 RPM overnight (12 h). The next morning 3 ml of the starter culture was inoculated into 500 ml of LB media containing 35 μg/ml of kanamycin and shaken at 37 °C and 200 RPM until the OD_600_ = 0.6. Protein expression was then induced by adding 0.5 mM IPTG and incubating at 25 °C. After 12 h, the cells were pelleted by centrifugation at 21,000*g* for 25 min. The pelleted cells were flash frozen in liquid nitrogen and stored at −80 °C until use.

Initially, WT and W197L/Y202N/M206V rPRMT1 were expressed and purified using our batch binding protocol (65), with previously described modifications (59). However, when we began working with the single mutants, we found that these constructs purified with a large amount of A260 contamination (260/280 ∼ 2). Analysis of this protein using Urea PAGE with SYBR Gold staining identified at least part of the contaminant as nucleic acid. We found that this contaminant would slowly dissociate over time, and we revised our purification protocol to exploit this dissociation to remove the contaminant.

To purify the PRMT1 constructs, 3 g of frozen cells was resuspended in lysis buffer (50 mM sodium phosphate, 20 mM imidazole, pH = 7.6) at a ratio of 20 ml buffer/g of cells. Cells were lysed by sonication and the lysate was pelleted at 41,000*g*. The soluble fraction was loaded onto a Cytiva 1 ml HisTrap Fast Flow column that had been equilibrated with lysis buffer. The column was washed in a high-salt wash buffer (50 mM sodium phosphate, 1 mM DTT, 1 mM EDTA, 1 M NaCl, pH = 7.6) for 30 ml, washed in a no-salt wash buffer (50 mM sodium phosphate, 1 mM DTT, 1 mM EDTA, pH = 7.6) for 144 ml, then washed with a tris wash buffer (20 mM tris, 1 mM EDTA, 1 mM DTT, pH = 7.6) for 10 ml. Proteins were eluted with a stepped imidazole gradient. Fractions were pooled based on purity assessed by the A260/280 ratio and by SDS-PAGE.

Pooled fractions were dialyzed in 50 mM sodium phosphate, 1 mM EDTA, 1 mM DTT, 10% glycerol, 0.1% Tween 20, pH = 7.6. We found that inclusion of a small amount of detergent in the dialysis buffer was necessary to prevent the single mutants from sticking to membranes in downstream processes. After dialysis, the samples were concentrated in an Amicon Ultra 10 kDa centrifugal concentrator. After the samples were concentrated, they were either used immediately in an activity assay or native PAGE analysis or beaded on liquid nitrogen and stored at −80 °C. This protocol typically yields ∼300 μl at ∼3 μM for the single mutants with high purity assessed by SDS-PAGE and an A260/280 ratio between 0.8 and 1. For WT the yield is typically ∼500 μl at ∼30 μM with high purity assessed by SDS-PAGE and an A260/A280 ratio of 0.6.

### Analytical ultracentrifugation

Sedimentation velocity (SV) experiments were performed in a Beckman ProteomeLab XL-I analytical ultracentrifuge equipped with scanning optics using an 8-hole rotor, 12 mm carbon-filled epoxy double-sector centerpieces, and quartz windows. Proteins to be analyzed were dialyzed in 50 mM sodium phosphate, 2 mM EDTA, pH 7.6 overnight at 4 °C. Samples were prepared containing 3 μM protein in the same buffer used for dialysis. 2 mM DTT was added to appropriate tubes and samples were incubated on ice 45 min, then filtered with a 0.22 μm filter prior to loading into the cell. Prepared cells were placed in the 8-hole rotor, and temperature equilibrated at 20 °C while resting under vacuum in the rotor chamber. SV scans were carried out at a rotor speed of 40,000 rpm while recording absorbance at 280 nm. Buffer viscosity, protein partial specific volumes, and fractional ratios were calculated using the software Sednterp (95). All SV data analysis was performed using the program Sedfit (96). Differential sedimentation coefficient distributions were calculated by least-squares boundary modeling of sedimentation velocity data using the continuous distribution c(s) Lamm equation model.

### Native PAGE

Either frozen or fresh protein was desalted using a Zeba Micro Spin Desalting Column, 7K MWCO using the manufacturer’s buffer exchange protocol. The buffer used for exchange was 50 mM sodium phosphate, 1 mM EDTA, 10% glycerol. Protein concentration was determined using A280 absorbance and the predicted extinction coefficients of each construct (97). The buffer exchanged samples were used to prepare samples at varying protein concentrations in native sample buffer (varying protein concentration, 50 mM sodium phosphate, 1 mM EDTA, 1 mM DTT, 10% glycerol, pH 7.6). Samples were incubated on ice for 20 min and then centrifuged at 21,000*g* to remove any aggregated protein prior to loading onto the gel. Prior to protein loading, 4 to 20% Mini-PROTEAN TGX gels were prerun at 40 V for 30 min at 4 °C using native running buffer (25 mM Tris Base, 192 mM glycine) in both the upper and lower chambers of the gel box, with the gel box itself cooled in an ice/water bath. Ten microliters of each sample was loaded onto the gel, and the gel was run at 40 V for an additional 30 min before the voltage was increased to 100 V, and the gel was run for 12 to 16 h to ensure separation of monomeric and dimeric PRMT1.

PRMT1 constructs were detected by western blotting using a 1° antibody solution (1:8000 rabbit α-PRMT1 [E5A8F from Cell Signaling]) in 5% BSA and TTBS and a 2° antibody solution (1:100,000 α-rabbit IgG HRP-linked antibody [7074S from Cell Signaling]) in 5% BSA and TTBS. The blot was treated with ECL reagent (Amersham ECL Select Western Blotting Detection reagent, RPN2235) and imaged. For gels with higher PRMT1 concentrations (>750 mM), the sensitivity of the α-PRMT1 antibody was not required. In these cases, an α-His HRP-linked antibody was used (1:1000 α-His HRP [Sc-8036 from Santa Cruz Biotechnology] in 5% NFDM and TTBS).

### Assessment of PRMT1 methyltransferase activity

PRMT1 activity was assessed using our previously described protocol (62, 66). Briefly, either fresh or frozen protein beads were desalted as described for the native PAGE to remove Tween 20. Enzyme activity was assessed at concentrations between 200 nM and 2 μM in assay buffer (final concentrations: 50 mM sodium phosphate pH = 7.6, 0.38 μM BSA, 10 nM AdoHcy nucleosidase, 1 mM DTT, 1 mM EDTA). In total, 2 μM AdoMet (1 μM ^3^[H] AdoMet) was added and the sample was equilibrated for 3 min at 37 °C, and the reaction was initiated by the addition of 200 μM R3 peptide substrate (Acetyl-GGRGGFGGRGGFGGRGGFGGK, biotin conjugated to C-terminal lysine). Five microliter samples were removed from the reaction at indicated timepoints and mixed with 6 μl of quench buffer (8 M guanidinium HCl, 2.5% TFA) to stop the reaction. Unreacted AdoMet was removed from the labeled peptide in each quenched sample using a C_18_ Zip Tip and the labeled product was quantified by scintillation counting. Substrate-blank reactions (to detect and account for low levels of automethylation sometimes observed) were run for each reaction condition and the signal from each substrate-blank sample was subtracted from the assay signal for the corresponding sample. In all cases the signal for the substrate-blank did not increase over time. Each reaction was done in duplicate and the data points are shown as the average ±standard deviation for each duplicate set. Where line fits are shown, the fits are to a linear model, which was applied only to the points through which the line is drawn.

### UV cross-linking

Frozen WT, W197L/Y202N/M206V, W197L, Y202N, and M206V rPRMT1 samples were buffer exchanged as described for native PAGE. Desalted proteins were diluted in the cross-linking buffer (final concentrations: 750 nM enzyme, 3.25 μM ^3^[H] AdoMet, 0.38 μM BSA, 1 mM DTT, 1 mM EDTA, 100 mM sodium phosphate pH = 7.6, final volume: 18 μl) in a clear 1.5 ml microcentrifuge tube and incubated at 37 °C for 10 min. The samples were then irradiated for 30 min at 37 °C using a 254 nm Mineralight Lamp Model UVG-11 with the opening of the sample tube held against the surface of the lamp directly below the bulb. Samples were quenched by boiling in 4X SDS sample buffer. In order to confirm that the cross-linked AdoMet was bound specifically, 100 μM unlabeled AdoMet was added to the cross-linking buffer of a duplicate WT sample. Samples were run on 12% polyacrylamide gels, transferred to a membrane, dried, then exposed to a phosphor screen for 2 months. The screen was imaged using a typhoon scanner. The dried membrane was then rehydrated in 70% methanol, rinsed with TBS, and blotted as described for native PAGE using the α-His antibody.

### Circular dichroism measurements

Frozen WT and W197L/Y202N/M206V samples were buffer exchanged as described for native PAGE using CD sample buffer (50 mM sodium phosphate, 1 mM DTT, pH = 7.6) and diluted to 0.5 mg/ml. CD data was collected in a Jasco J-1500 CD spectropolarimeter with a data pitch of 0.5 nm, a scanning speed of 100 nm/min, and a Digital Integration Time of 4 s. Background signal for all runs was collected using the CD sample buffer and was subtracted from the sample signal. Data was normalized to mean residue ellipticity. All samples were done in duplicate, with representative spectra shown.

### Computational enzyme preparation

A 2.35 Å resolution crystal structure of PRMT1 (PDB ID: 1OR8) (44) was used to generate the initial Cartesian coordinates for MD simulations. Adopting a protocol from our previous study (57) the missing residues (residues 26–40) of the N-terminal region that forms the active site were added in using the comparative modeling program MODELLER 9.10 (98). The BOMB (99) software was used to grow the Arg-containing peptide (GGRG) into the active site. The cocrystallized AdoHcy was methylated to yield (*S*,*S*)-AdoMet. Mutations to the PRMT1 active site residues were made using the Yasara software (100). The tleap module of AmberTools16 was used to add hydrogen atoms into the PRMT1 model.

### Molecular dynamics simulation

The WT and mutant PRMT1 monomer enzyme/substrate complexes were then subjected to MD simulations. The enzyme complexes were solvated explicitly using a TIP3P (101) orthorhombic water box that extended 10 Å away from the protein, and the overall charge of the system was neutralized by adding a suitable number of sodium cations. The generalized Amber force field (GAFF) (102) was used to parameterize AdoMet, and the protein topology file was created using the ff14SB force field (103). All simulations were carried out using the GPU-enabled Amber16 pmemd engine (104, 105). The water molecules and Na^+^ ions were exclusively minimized in the initial structures using the conjugate gradient (CG) method for 3000 steps, followed by 10,000 steps of CG minimization for the entire system. Thereafter, the system was gradually heated from 0 to 300 K using a constant NVT ensemble over 50 ps with a weak-coupling algorithm and temperature coupling value of 2.8 ps. To correct the density of the system, a 500 ps simulation was performed using a constant NPT ensemble at 300 K and 1 atm with the temperature and pressure coupling values set to 2.0 ps. The system was then switched back to the NVT ensemble and further equilibrated for 500 ps. Following the minimization and equilibration phase, a 10 ns NVT production run using unbiased MD was carried out to compute boost potentials for the subsequent 1000 ns aMD simulations (67, 106, 107). Degrees of freedom boosted per system and their respective values are provided in Table S1. In all MD simulations long-range electrostatics were accounted for by using the particle mesh Ewald, all covalent bonds involving hydrogen atoms were constrained with the SHAKE algorithm, periodic boundary conditions were enforced using a nonbonded cutoff distance of 12 Å, and a time step of 1.0 fs was utilized. Analysis was performed with the cpptraj and ptraj programs available in the AmberTools16 suite (108). The root-mean-square deviations (RMSDs) were calculated to monitor the structural stability of each simulation, and the RMSD values of the backbone protein atoms are provided in Figure S1.

### Computational clustering analysis

Clustering analysis is a method of determining a population ensemble during MD simulation by grouping similar conformations together over the trajectory. In this study the cpptraj “average-linkage” algorithm was used (75, 108, 109). In the distance metric, RMSD of atoms with a sieve of 10 was applied. Finally, the average of all frames in each cluster was printed out as an output coordinate in pdb format, which are provided in the supporting information.

## Data availability

All data are contained within the manuscript.

## Supporting information

This article contains supporting information.

## Conflict of interest

The authors declare that they have no conflicts of interest with the contents of this article.
