# Supplementary material for: Naturally occurring cancer-associated mutations disrupt oligomerization and activity of protein arginine methyltransferase 1 (PRMT1)
Source: J Biol Chem. 2021 Oct 22;297(5):101336. doi: 10.1016/j.jbc.2021.101336 (PMC8592882; doi:10.1016/j.jbc.2021.101336)
Supplement: Figure S1 and Table S1 [file mmc1.docx]

**SUPPORTING INFORMATION**

Naturally occurring cancer-associated mutations disrupt oligomerization and activity in Protein Arginine Methyltransferase 1 (PRMT1)

**Owen M Price^1^**^‡^**, Abhishek Thakur^2^**^‡^**, Ariana Ortolano^1^, Arianna Towne^1^, Caroline Velez^2^, Orlando Acevedo^2^, and Joan M. Hevel^1*^**

^1^Department of Chemistry and Biochemistry, Utah State University, Logan, UT, USA

^2^Department of Chemistry, University of Miami, Coral Gables, FL, USA

^‡^These authors contributed equally to the studies

**LIST OF MATERIAL INCLUDED:**

**Figure S1**: Root-mean-square deviations (RMSDs) relative to the first frame for the mutant PRMT1 protein backbone atoms (N, Cα, and C

**Table S1:** Accelerated Molecular Dynamics Boost Potentials used in the WT and Mutant PRMT1 Simulations

**Zip file:** A .zip file containing PDB structures of the top 3 PRMT1 dimerization arm variants


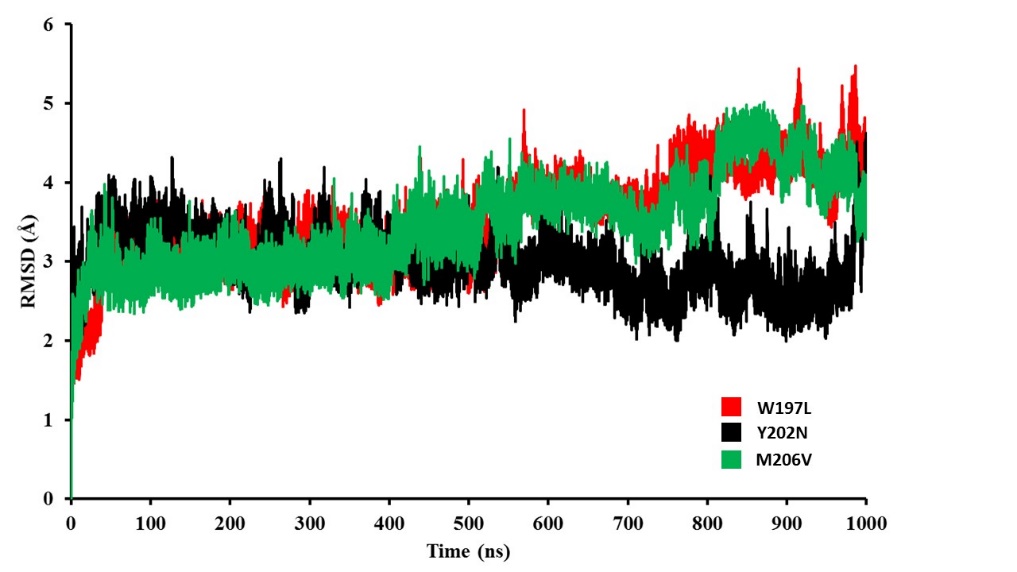


**Figure S1**: Root-mean-square deviations (RMSDs) relative to the first frame for the mutant PRMT1 protein backbone atoms (N, Cα, and C).

**Table S1: Accelerated Molecular Dynamics Boost Potentials used in the WT and Mutant PRMT1 Simulations**

| **System** | **alphaD** | **alphaP** | **EthreshP** | **EthreshD** |
| --- | --- | --- | --- | --- |
| W197L | 234 | 9361 | -132486 | 5338 |
| Y202N | 234 | 9362 | -132484 | 5337 |
| M206V | 234 | 9362 | -132452 | 5347 |
